# Case Report: Synchronous colorectal adenocarcinomas with discordant mismatch repair status: a case of lynch-like syndrome and serrated pathway association

**DOI:** 10.3389/fonc.2026.1770875

**Published:** 2026-04-27

**Authors:** Daming Chen, Jiansheng Zhang, Jinchao Bi, Zhiyue Bai, Lei Zhang, Jingzhen Bai

**Affiliations:** 1 Department of General Surgery, Tianjin Baodi Hospital, Affiliated Baodi Hospital of Tianjin Medical University, Tianjin, China; 2 Department of Pathology, Tianjin Baodi Hospital, Affiliated Baodi Hospital of Tianjin Medical University, Tianjin, China; 3 Medical Record Department, Tianjin Baodi Hospital, Affiliated Baodi Hospital of Tianjin Medical University, Tianjin, China

**Keywords:** synchronous colorectal cancer, mismatch repair deficiency, lynch-like syndrome, serrated pathway, regional metastatic lymph node

## Abstract

**Objective:**

Synchronous colorectal cancers (SCRCs) with discordant mismatch repair (MMR) status present unique clinical and therapeutic challenges. This case report describes a rare instance of synchronous ascending colon and rectal adenocarcinomas arising from distinct tumorigenic pathways (Lynch-like syndrome and serrated pathway). We aim to explore the mechanism underlying MMR status heterogeneity, and emphasize the clinical value of lesion-specific molecular profiling combined with regional metastatic lymph node MMR phenotyping for individualized treatment of this condition.

**Methods:**

A 67-year-old female with secondary peritonitis was enrolled. The patient underwent sequential imaging, colonoscopy, and histopathological examination, which confirmed synchronous double primary colorectal adenocarcinomas. Systematic molecular analyses were performed, including MMR immunohistochemistry (IHC), BRAF V600E mutation testing, germline next-generation sequencing (NGS) targeting MMR-related genes, and MLH1 promoter methylation testing via enzyme conversion NGS. MMR phenotyping of regional metastatic lymph nodes was performed to assess the invasive potential of each lesion and guide adjuvant treatment. The patient received adjuvant CAPEOX-based chemoradiotherapy and was followed up for 24 months.

**Results:**

Histopathology confirmed two synchronous primary adenocarcinomas: the ascending colon tumor was poorly differentiated adenocarcinoma with deficient MMR (dMMR; MLH1-, PMS2-; MSH2+, MSH6+), BRAF V600E wild-type, and negative MLH1 promoter methylation in both tumor tissue and peripheral blood; the rectal tumor was moderately-to-poorly differentiated adenocarcinoma with proficient MMR (pMMR; MLH1+, PMS2+, MSH2+, MSH6+), adjacent to a sessile serrated lesion (SSL). Germline NGS revealed no pathogenic MMR gene variants, consistent with a diagnosis of Lynch-like syndrome (LLS). Regional metastatic lymph nodes showed a pMMR phenotype, consistent with the rectal primary tumor, indicating higher metastatic potential of the rectal lesion. No recurrence or metastasis was observed at the 24-month follow-up, with satisfactory patient quality of life.

**Conclusion:**

Lesion-specific molecular characterization combined with regional lymph node MMR phenotyping is critical for the precise management of SCRCs with discordant MMR status. This case provides a referable diagnostic workflow and surgical decision-making framework for this rare clinical scenario, supporting risk-adapted individualized therapy for molecularly heterogeneous colorectal cancer.

## Introduction

Colorectal cancer (CRC) is one of the leading causes of cancer-related mortality worldwide, with adenocarcinoma as the most prevalent subtype (1). DNA mismatch repair (MMR) status has been established as a core biomarker for prognostic stratification and therapeutic guidance in CRC: proficient MMR (pMMR) accounts for approximately 85% of all CRC cases, while deficient MMR (dMMR) accounts for the remaining 15% (2, 3). Therapeutically, dMMR tumors exhibit high immunogenicity and superior response to immune checkpoint inhibitors, whereas pMMR tumors have limited immunotherapy response and mainly rely on conventional chemoradiotherapy (4–6).

Synchronous colorectal cancers (SCRCs), defined as two or more independent primary colorectal adenocarcinomas detected simultaneously or within 6 months of the first diagnosis, account for 2%-4% of all CRC cases (7, 8). Notably, synchronous colorectal cancers (SCRCs) can present with discordant mismatch repair (MMR) status between paired primary lesions, with the reported incidence reaching 18.5% in Western cohorts (nearly consistent with the pooled rate of 18.2%), and a 5.1% incidence observed in an East Asian cohort. If molecular testing is performed on only a single lesion of SCRCs, approximately 23% of patients will face erroneous treatment decisions, due to the missed intertumoral heterogeneity of both MMR status and key therapeutic biomarkers including KRAS and BRAF mutations (9, 10). This inter-lesion heterogeneity poses significant diagnostic and therapeutic dilemmas in clinical practice, especially when the lesions arise from distinct tumorigenic pathways.

Lynch-like syndrome (LLS), defined as dMMR CRC without germline pathogenic MMR mutations, negative BRAF V600E mutation, and negative MLH1 promoter methylation, remains a diagnostic and management challenge in clinical practice (11). Meanwhile, synchronous occurrence of an LLS-associated dMMR tumor and a serrated pathway-related pMMR tumor in the same patient is extremely rare, with no unified standardized diagnostic workflow in current guidelines. Herein, we present a rare case of synchronous double primary CRCs arising from independent LLS and serrated pathways, to detail the diagnostic workflow, surgical decision-making, and clinical value of lymph node MMR phenotyping for this rare clinical scenario.

## Case presentation

### Patient information and clinical history

A 67-year-old female farmer presented to our emergency department with a 15-day history of persistent abdominal pain and bloating, aggravated with fever for 1 day. The abdominal pain was mainly localized in the middle and lower abdomen, presenting as paroxysmal colic that aggravated after eating and was slightly relieved after defecation, with a maximum body temperature of 38.6°C on admission. The patient had no significant past medical history, no smoking or alcohol consumption, and no family history of malignant tumors or hereditary gastrointestinal diseases. She was married, had one healthy son with good family support, and had lost approximately 4kg of body weight in the month prior to admission.

On physical examination, the patient had a body temperature of 38.2°C, pulse of 98 beats/min, blood pressure of 132/86mmHg, and blood oxygen saturation of 98% without oxygen inhalation. The abdomen was flat, with mild muscle tension, diffuse abdominal tenderness (most prominent in the middle and lower abdomen) with rebound tenderness, and weakened bowel sounds (1–2 times/min). Digital rectal examination identified a circumferential hard mass with poor mobility, estimated at 9 cm from the anal verge, with fresh blood staining on the finger cot after withdrawal. This location was subsequently confirmed by colonoscopy. No other abnormal findings were noted on systemic physical examination.

### Diagnostic assessment

Laboratory tests on admission showed leukocytosis (white blood cell count 18.0×10^9^/L, neutrophil percentage 88.2%), and elevated tumor markers: carcinoembryonic antigen (CEA) 12ng/mL (reference range 0-5ng/mL), carbohydrate antigen 125 (CA125) 74.2U/mL (reference range 0-35U/mL). Liver and kidney function, electrolytes, and coagulation function were within normal limits.

**Figure 1:**
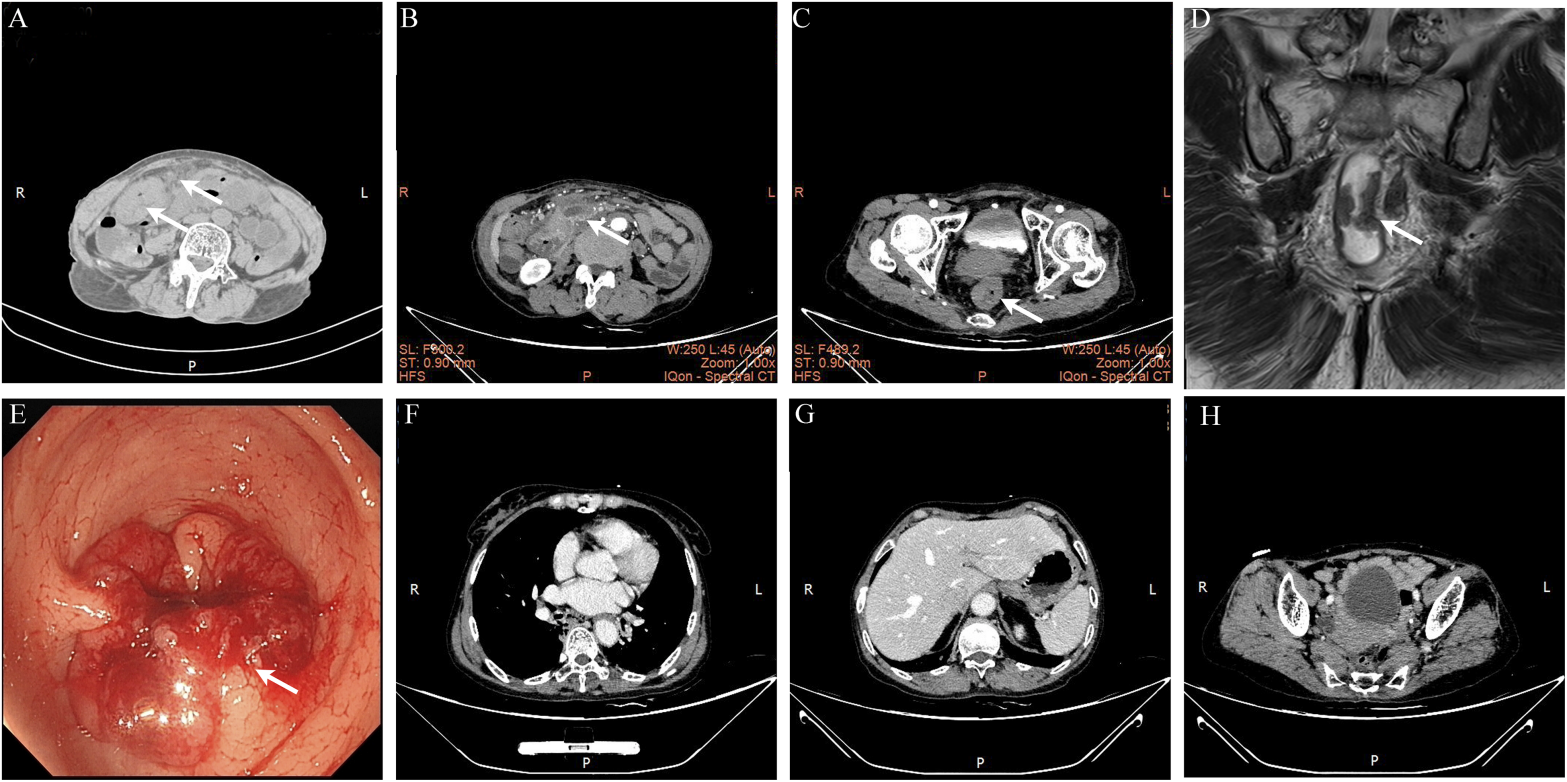
Imaging and colonoscopic findings. **(A)** Emergency non-contrast abdominopelvic CT, with arrows indicating the ascending colon mass and abdominal inflammatory changes; **(B)** Contrast-enhanced abdominopelvic CT, with an arrow indicating the ascending colon mass; **(C)** Contrast-enhanced abdominopelvic CT, with an arrow indicating the rectal mass; **(D)** T2-weighted high-resolution pelvic MRI showing the rectal mass; **(E)** Colonoscopy image, with an arrow indicating the severe intestinal stenosis caused by the rectal mass; **(F-H)** Surveillance thoracoabdominal CT at 24-month follow-up, showing no evidence of tumor recurrence or metastasis.

Emergency non-contrast abdominopelvic CT showed thickening of the ascending colon and rectal wall with surrounding fat infiltration and enlarged lymph nodes, extensive abdominopelvic fat space turbidity with pelvic effusion, consistent with secondary peritonitis; no free air or distant organ metastasis was observed (**Figure 1A**).

After 4 days of intravenous antibiotic anti-infection therapy, the patient’s peritonitis signs were completely relieved. Subsequent thoracoabdominal enhanced CT and high-resolution rectal MRI confirmed the ascending colon and rectal space-occupying lesions, with no pulmonary, hepatic, or osseous metastasis; the preoperative clinical stage was evaluated as cT3N1M0 for both lesions (**Figures 1B–D**).

Emergency colonoscopy was performed to 9cm from the anal verge, where a circumferential ulcerative rectal mass was identified, causing severe intestinal stenosis that prevented further advancement of the endoscope. Biopsy of the rectal mass confirmed moderately-to-poorly differentiated adenocarcinoma (**Figure 1E**).

### Surgical treatment and pathological findings

The patient’s secondary peritonitis was attributed to transmural inflammatory infiltration of the two primary tumors rather than free intestinal perforation. After admission, she received 4 days of standardized intravenous antibiotic therapy, with complete resolution of systemic inflammatory and abdominal peritonitis signs before surgery. Subsequently, the patient underwent laparoscopic total colectomy, anterior rectal resection, D3 lymphadenectomy, and ileal J-pouch rectal anastomosis. Intraoperative exploration confirmed marked inflammatory fibrinous adhesions and focal exudation around both the ascending colon and rectal tumors, which was consistent with the subacute inflammatory process secondary to tumor transmural infiltration over the 7-day disease course; no evidence of free intestinal perforation, diffuse purulent peritonitis, or intestinal wall necrosis was identified. Meanwhile, residual mild-to-moderate inflammatory edema of the colonic wall was noted intraoperatively, and such a state is associated with a significantly elevated risk of postoperative anastomotic leakage for any colonic anastomosis, which further supported the decision of total colectomy to avoid high-risk colonic anastomoses and retain only one safe ileal J-pouch rectal anastomosis. The inflammatory adhesions were safely and completely lysed, and the residual rectal stump showed satisfactory blood supply and intact wall texture, ensuring safe anastomosis. The operation was uneventful, and the patient recovered smoothly without any perioperative complications.

**Figure 2:**
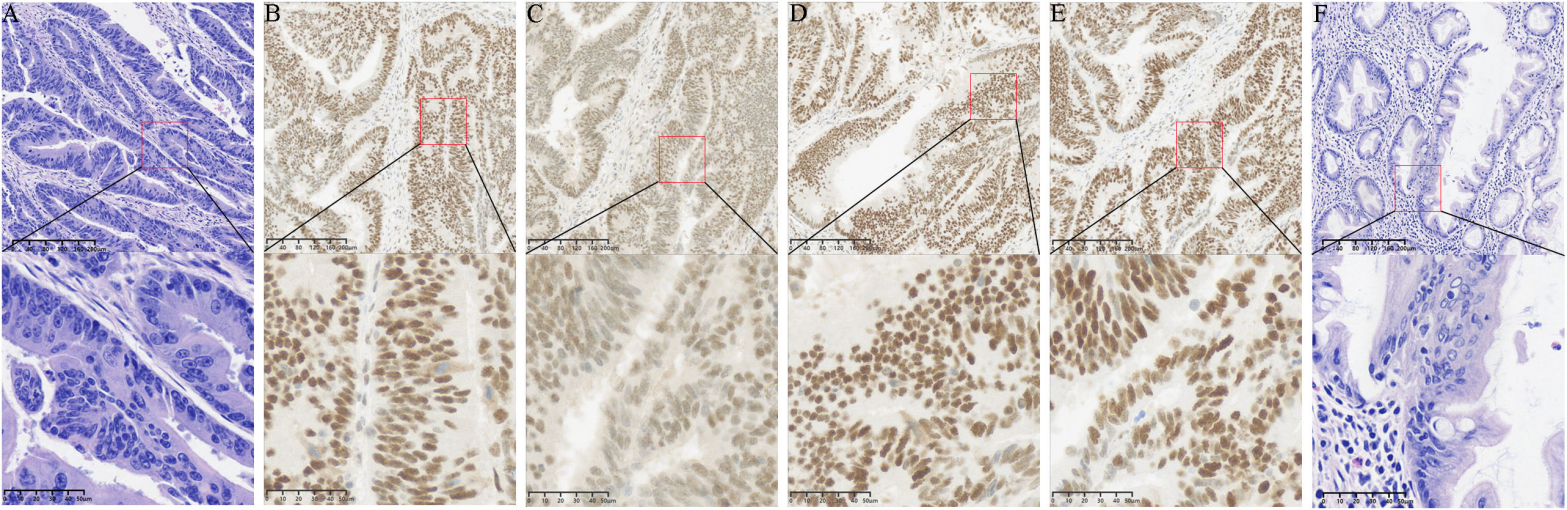
Pathological features of the rectal adenocarcinoma and adjacent SSL. **(A)** Hematoxylin and eosin (H&E) staining of the moderately-to-poorly differentiated rectal adenocarcinoma; **(B–E)** IHC staining of the rectal tumor: **(B)** MLH1 (positive), **(C)** PMS2 (positive), **(D)** MSH2 (positive), **(E)** MSH6 (positive); **(F)** H&E staining of the adjacent sessile serrated lesion (SSL). All micrographs were acquired at 300 dpi resolution. Upper panels: original magnification ×100, scale bar = 40 μm; lower panels: original magnification ×400, scale bar = 10 μm.

Postoperative paraffin pathology confirmed two synchronous primary adenocarcinomas. The rectal lesion was a moderately-to-poorly differentiated adenocarcinoma, with a pathological T stage of pT3: the tumor infiltrated through the muscularis propria into the perirectal mesorectal fat. On pathological examination, the rectal lesion showed positive lymphovascular and perineural invasion, with 2 out of 12 regional lymph nodes positive for metastatic carcinoma. IHC showed a pMMR phenotype with retained expression of MLH1, PMS2, MSH2 and MSH6, and a Ki-67 proliferation index of 80%; an adjacent sessile serrated lesion (SSL) was observed beside the rectal tumor. Pathological features of the rectal adenocarcinoma and adjacent SSL are presented in **Figure 2**.

**Figure 3:**
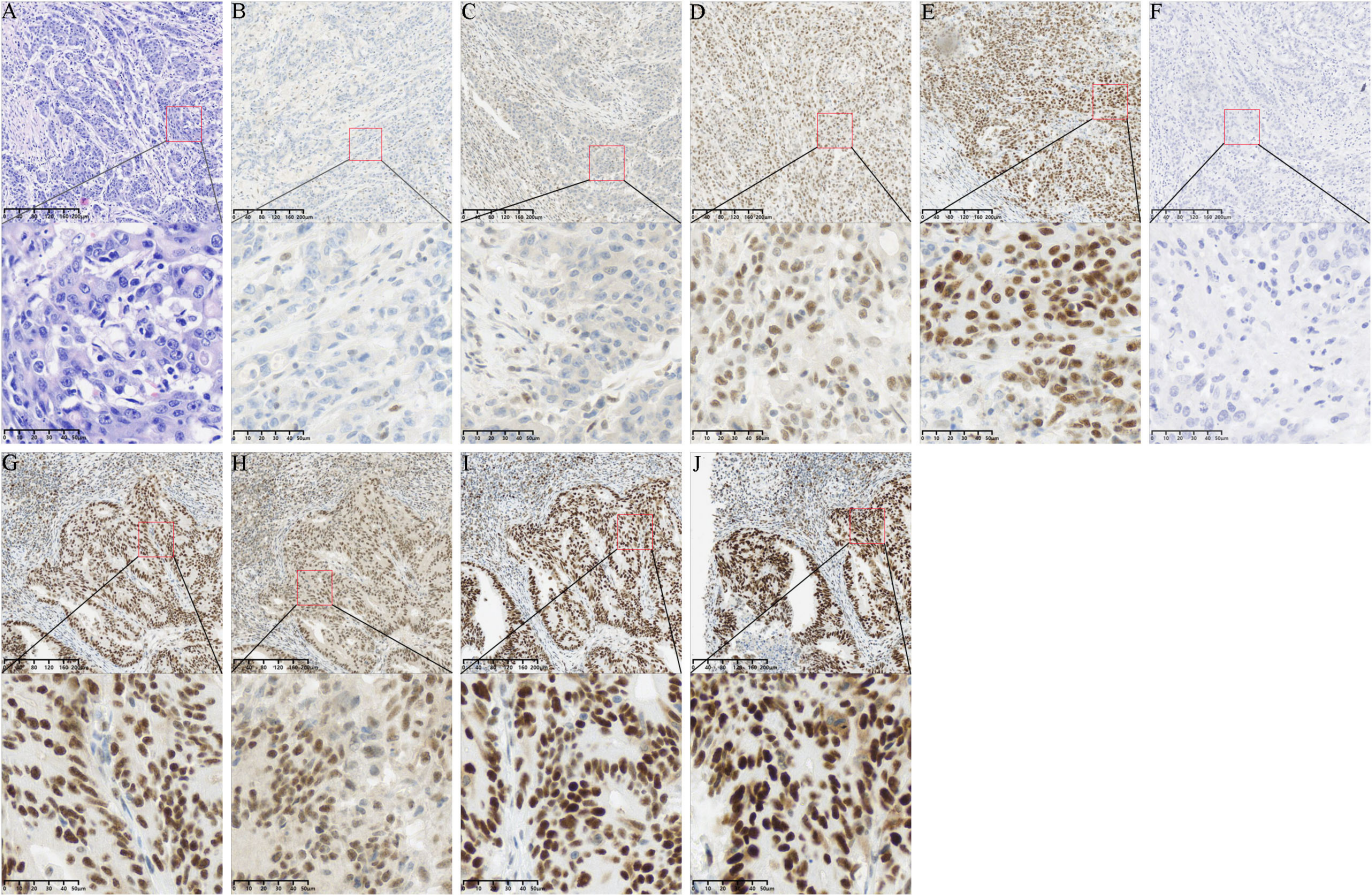
Pathological and molecular features of the ascending colon adenocarcinoma and regional metastatic lymph node. **(A)** H&E staining of the poorly differentiated ascending colon adenocarcinoma; **(B–F)** IHC staining of the ascending colon tumor: **(B)** MLH1 (negative), **(C)** PMS2 (negative), **(D)** MSH2 (positive), **(E)** MSH6 (positive), **(F)** BRAF V600E (wild-type, negative); **(G–J)** IHC staining of the regional metastatic mesenteric lymph node: **(G)** MLH1 (positive), **(H)** PMS2 (positive), **(I)** MSH2 (positive), **(J)** MSH6 (positive). All micrographs were acquired at 300 dpi resolution. Upper panels: original magnification ×100, scale bar = 40 μm; lower panels: original magnification ×400, scale bar = 10 μm.

The ascending colon lesion was a poorly differentiated adenocarcinoma, with a pathological T stage of pT3: the tumor infiltrated through the muscularis propria into the subserosal layer and pericolonic adipose tissue. On pathological examination, the ascending colon lesion showed negative lymphovascular and perineural invasion, with 0 out of 12 regional lymph nodes positive for metastasis. IHC showed a dMMR phenotype with complete loss of MLH1 and PMS2 expression, retained MSH2 and MSH6 expression, and a Ki-67 proliferation index of 90%; BRAF V600E was wild-type, and MLH1 promoter methylation testing in the tumor tissue was negative (**Figures 3A–F**).

According to the 8th edition of the American Joint Committee on Cancer (AJCC) TNM staging system for colorectal cancer, the final postoperative pathological stage of the synchronous double primary colorectal carcinomas was pT3N1aM0 (stage IIIB), based on the highest T stage and highest N stage of the two independent primary lesions.

IHC of the regional metastatic lymph nodes showed a pMMR phenotype, completely consistent with the rectal primary tumor (**Figures 3G–J**). Germline NGS targeting MLH1, MSH2, MSH6, and PMS2 genes revealed no pathogenic or likely pathogenic variants, and peripheral blood MLH1 promoter methylation testing was negative, which was fully consistent with the established diagnostic criteria of LLS.

### Postoperative treatment and follow-up

The patient received 6 cycles of adjuvant CAPEOX chemotherapy (capecitabine 1000 mg/m² orally twice daily on days 1–14, oxaliplatin 130 mg/m² intravenously on day 1, every 21 days) combined with concurrent pelvic intensity-modulated radiotherapy (50.4 Gy in 28 fractions). Only grade 1 hand-foot syndrome occurred during treatment, which was relieved with symptomatic management, with no treatment interruption.

**Figure 4:**
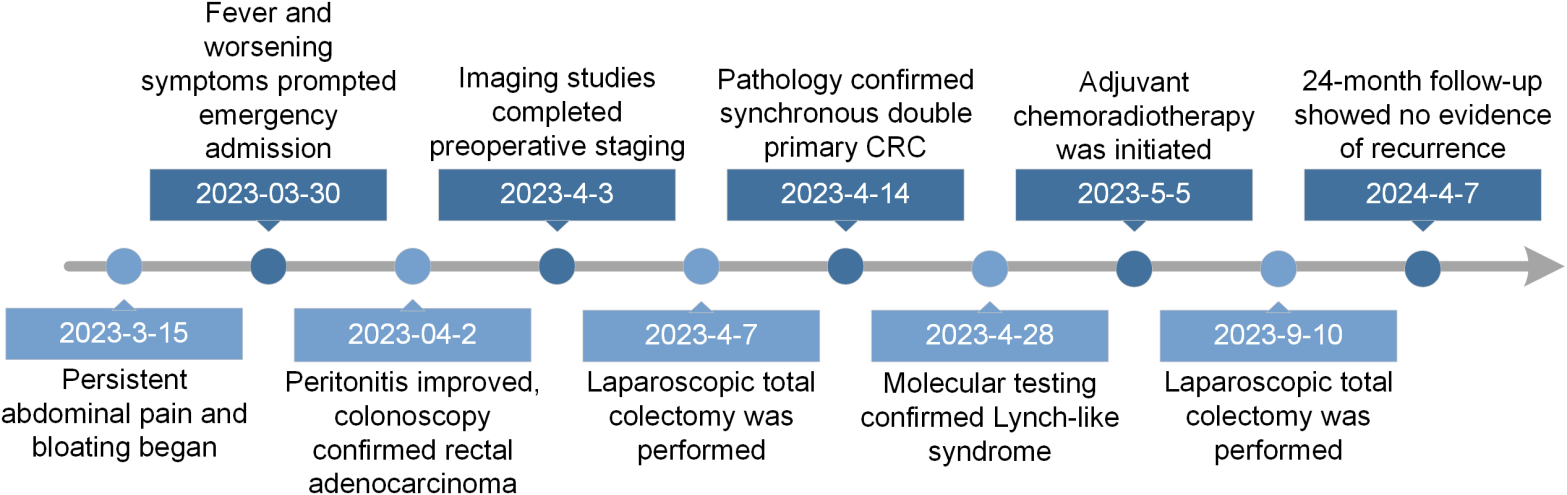
Timeline of key clinical events. The patient developed persistent abdominal pain and bloating on March 15, 2023. Fever and worsening symptoms prompted emergency admission on March 30, 2023. Peritonitis improved and colonoscopy confirmed rectal adenocarcinoma on April 2, 2023. Imaging studies completed preoperative staging on April 3, 2023. Laparoscopic total colectomy was performed on April 7, 2023. Pathology confirmed synchronous double primary colorectal cancer on April 14, 2023. Molecular testing confirmed Lynch-like syndrome on April 28, 2023. Adjuvant chemoradiotherapy was initiated on May 5, 2023, and completed on September 10, 2023. The 24-month follow-up showed no evidence of recurrence on April 7, 2025.

The patient was followed up every 6 months with regular thoracoabdominal CT and tumor marker re-examination. At the 24-month comprehensive follow-up, the patient had no tumor recurrence or metastasis, with good recovery of defecation function, satisfactory self-assessed quality of life, and an EQ-5D score of 85/100. Surveillance imaging at the 24-month follow-up showed no evidence of tumor recurrence or metastasis (**Figures 1F–H**). The full timeline of the patient’s diagnosis, treatment, and follow-up is summarized in **Figure 4**.

### Patient perspective

When the abdominal pain started, I thought it was just a common stomach issue and never expected it to be cancer. I was extremely scared when the doctors told me there were two tumors in my bowel, worried about not surviving the surgery and the burden on my family. The medical team explained the treatment plan and risks to me and my son in detail before the operation, which made me feel much more at ease, and I fully cooperated with all the treatment.

In the first 3 months after surgery, I had 5–6 bowel movements a day, which brought a lot of inconvenience to my life. But as time went on, my bowel function gradually recovered, and it returned to 3–4 times a day 6 months after the operation, with no fecal incontinence. I completed all the chemotherapy and radiotherapy successfully, with only slight numbness in my hands and feet and no other serious discomfort.

Now 2 years after the operation, I have returned to my normal daily life and housework, and I am very satisfied with the treatment effect. After learning about the Lynch-like syndrome diagnosis and the potential familial cancer risk, I took the initiative to ask my son and siblings to have regular colorectal cancer screening, and I strictly follow the doctor’s advice for regular re-examinations every 6 months. I am very grateful to the medical team for their careful treatment, and I am willing to share my case to help more patients with the same disease.

## Discussion

This case presents a rare clinical scenario of synchronous double primary CRCs with completely discordant MMR status, derived from independent LLS and serrated tumorigenic pathways. We systematically analyzed the mechanistic basis of inter-lesion MMR heterogeneity, standardized diagnostic workflow, and clinical management strategy for this rare disease entity, to provide a practical reference for clinical practice.

### Mechanistic basis of MMR status heterogeneity in synchronous CRCs

The core feature of this case is the coexistence of two independent primary CRCs with discordant MMR status, which can be systematically explained by two complementary mechanistic frameworks. First, the patient may have unrecognized low-penetrance germline variants in cancer susceptibility genes (such as MUTYH, ATM), leading to decreased genomic stability of the entire colorectal mucosa and overall increased tumorigenic susceptibility (12–14). On this basis, the ascending colon and rectal mucosa underwent completely independent somatic second-hit events: the ascending colon tumor acquired somatic biallelic inactivation of MMR genes leading to a dMMR phenotype, while the rectal tumor developed driver events related to the serrated pathway maintaining a pMMR phenotype. This mechanism is strongly supported by the study of Khandakar et al., which reported that even patients with classic Lynch syndrome can develop pMMR CRCs due to distinct somatic second-hit events (15).

Second, the two tumors originated from two completely independent and parallel carcinogenic pathways. For the ascending colon tumor, the dMMR phenotype, wild-type BRAF V600E, negative MLH1 promoter methylation, and absence of pathogenic germline MMR variants fully meet the diagnostic criteria of LLS (11). The most core mechanism of LLS is somatic biallelic inactivation of MMR genes, including somatic mutations and loss of heterozygosity (LOH) (16, 17). Limited by targeted panel testing without whole-genome sequencing, we cannot completely rule out other potential mechanisms, such as germline variants in non-MMR genes affecting the MMR system, or rare MMR gene variants missed by the panel (13, 18). For the rectal tumor, the anatomical coexistence with an SSL strongly indicates its origin from the serrated neoplasia pathway. Distal colorectal SSLs often progress to pMMR CRCs with wild-type BRAF, which is a rare but well-recognized subtype of the serrated pathway, highly consistent with the features of this case (19, 20).

### Standardized diagnostic workflow for LLS and discordant MMR SCRCs

Accurate etiological diagnosis of dMMR lesions is the core of clinical management for SCRCs with discordant MMR status. Based on this case and current clinical consensus, we summarize a 6-step standardized diagnostic workflow to address common clinical diagnostic pitfalls (1): Universal MMR testing for each synchronous CRC lesion to avoid missed detection of inter-lesion heterogeneity (2); BRAF V600E and MLH1 promoter methylation testing for dMMR lesions with MLH1/PMS2 loss to exclude sporadic dMMR CRC (3); Germline MMR gene testing to exclude classic Lynch syndrome (4); Confirmation of LLS diagnosis for eligible cases (5); MMR phenotyping of regional metastatic lymph nodes to clarify the clonal origin of metastasis (6); Formulation of individualized treatment and surveillance strategies based on the molecular characteristics of all lesions.

This workflow effectively addresses the critical diagnostic pitfall of treatment decision-making based on single-lesion MMR testing in synchronous/metachronous gastrointestinal carcinomas, which has been reported to result in a misdiagnosis rate of approximately 26% (nearly 1 in 4 cases) in clinical practice (9). It also standardizes the diagnostic process of LLS, which is consistent with the current consensus on LLS clinical management (21). In this case, we supplemented MLH1 promoter methylation testing in both tumor tissue and peripheral blood with negative results, which further improved the diagnostic accuracy of LLS.

### Clinical value of regional lymph node MMR phenotyping

Accurate identification of the metastatic source and risk stratification of primary lesions are the basis for adjuvant treatment decision-making in SCRCs. Traditional clinical practice usually infers the metastatic source only based on the anatomical location of regional lymph nodes, but this method has significant limitations. While anatomical location is routinely used to preliminarily infer the source of lymph node metastasis in clinical practice, skip lymph node metastasis does occur in pT3-T4 stage colorectal cancer, with reported incidence rates of 11.4% in pT3 disease and 12.6% in pT4 disease (22). This atypical lymphatic spread pattern breaks the conventional sequential lymph node metastasis rule, making it unreliable to solely rely on anatomical topography or imaging findings to determine the true origin of metastatic lymph nodes in locally advanced rectal cancer (23).

In this case, the pMMR phenotype of the metastatic lymph nodes is completely consistent with the rectal primary tumor, which provides direct molecular evidence that the regional metastasis is derived from the rectal lesion, excluding skip metastasis from the ascending colon tumor. More importantly, this test achieves accurate stratification of the invasive potential of the two lesions: the pMMR rectal tumor has stronger lymphatic metastatic ability and is the main source of postoperative recurrence risk. This is the core basis for our selection of adjuvant CAPEOX chemoradiotherapy, which targets the high-risk pMMR lesion and avoids unnecessary immunotherapy for the dMMR lesion. This fully demonstrates that regional lymph node MMR phenotyping is a key supplement to the traditional anatomical inference method for SCRCs with discordant MMR status, and should be recommended as a routine test for such cases.

### Rationality of total colectomy surgical decision-making

The selection of surgical procedure for SCRCs needs to balance radical tumor resection, perioperative safety, and long-term functional outcomes. For this patient, total colectomy was selected based on four core clinical considerations, which are fully compliant with current clinical guidelines. First, the two lesions are located in the ascending colon and rectum with a non-contiguous, dispersed distribution. Total colectomy achieves complete resection of all lesions, and this surgical strategy is recommended in the NCCN Clinical Practice Guidelines in Oncology for Colon Cancer (Version 1.2026) for patients with synchronous non-contiguous CRC (14). Second, the patient had secondary peritonitis preoperatively, and residual mild-to-moderate inflammatory edema of the colonic wall was still noted intraoperatively. Such persistent mucosal and mural changes induced by prior peritonitis can significantly elevate the risk of anastomotic leakage when any colonic anastomosis is performed (24, 25). Total colectomy only requires one ileal pouch-rectal anastomosis, while segmental resection of the ascending colon and rectum requires two digestive tract anastomoses, which significantly reduces the risk of anastomotic leakage in this high-risk context. Third, preoperative colonoscopy could not be completed due to severe intestinal stenosis caused by the rectal mass, which cannot rule out the presence of other space-occupying lesions in the colon that were not detected by CT and MRI (7). Total colectomy fundamentally eliminates the risk of missed synchronous lesions, which is a critical consideration for this patient. Fourth, patients with Lynch-like syndrome (LLS) have an approximately 2.1-fold increased risk of colorectal cancer compared with those with sporadic colorectal cancer (26). Total colectomy may reduce this risk and alleviate the burden of intensive colonoscopic surveillance (27). The patient and her family were fully informed of the advantages and disadvantages of different surgical strategies, and they actively requested total colectomy after adequate communication.

Regarding the timing of surgery, the patient had a preoperative clinical stage of cT3N1M0 colon cancer, for which upfront radical resection is the standard recommended approach according to the NCCN Clinical Practice Guidelines (14). More importantly, the patient had severe rectal stenosis with an extremely high risk of complete intestinal obstruction during neoadjuvant therapy, and the ascending colon lesion could not be biopsied preoperatively to clarify its molecular characteristics, making it impossible to formulate an optimal neoadjuvant regimen that takes into account both lesions. The postoperative pathological results confirmed the discordant MMR status of the two lesions, further verifying that upfront surgery followed by individualized adjuvant therapy is the most reasonable strategy for this patient.

We acknowledge that for isolated obstructing T3N1M0 rectal cancer, the standard of care is diversion with neoadjuvant therapy. However, this case presented synchronous double primary CRCs with discordant MMR status and secondary peritonitis, necessitating individualized surgical decision-making that deviated from standard protocols.

### Clinical significance and key takeaways

This case has important clinical reference value for the management of SCRCs with discordant MMR status. First, it reports a rare scenario of synchronous double primary CRCs derived from LLS and serrated pathways with discordant MMR status, which enriches the molecular phenotype spectrum of SCRCs and deepens the understanding of the biological heterogeneity of synchronous colorectal tumors. Second, it systematically elaborates the clinical value of regional lymph node MMR phenotyping for this disease entity, which provides a practical method for accurate risk stratification and adjuvant treatment decision-making in clinical practice. Third, it proposes a standardized diagnostic workflow and surgical decision-making framework for SCRCs with discordant MMR status, which provides a direct reference for the standardized management of similar rare cases.

## Limitations

This study has several limitations that need to be acknowledged. First, BRAF V600E testing was not performed on the SSL adjacent to the rectal tumor, so the direct molecular linkage between the SSL and the rectal adenocarcinoma cannot be confirmed. Second, only targeted germline panel testing for MMR genes was performed, and whole-genome sequencing of the tumor tissue was not performed, so the specific somatic molecular events of LLS (such as biallelic MMR gene mutations and LOH) cannot be clarified. Third, this is a single-center case report, and the conclusions need to be further verified by multi-center large-sample studies.

## Conclusion

This rare case of synchronous CRCs with discordant MMR status, derived from LLS and serrated pathways respectively, highlights the necessity of systematic lesion-specific molecular testing and regional metastatic lymph node MMR phenotyping in the management of molecularly heterogeneous CRC. This case provides a referable diagnostic workflow and surgical decision-making idea for the standardized diagnosis and treatment of SCRCs with discordant MMR status, and emphasizes the core value of individualized diagnosis and treatment for rare molecular phenotype CRC.

## Data Availability

The datasets presented in this study can be found in online repositories. The names of the repository/repositories and accession number(s) can be found in the article/**Supplementary Material**.
