# Supplementary material for: Case Report: Synchronous colorectal adenocarcinomas with discordant mismatch repair status: a case of lynch-like syndrome and serrated pathway association
Source: Front Oncol. 2026 Apr 27;16:1770875. doi: 10.3389/fonc.2026.1770875 (PMC13158114; doi:10.3389/fonc.2026.1770875)
Supplement: Supplementary file 1 [file DataSheet1.pdf]

CARE 2020 Checklist for Case Report

Manuscript Basic Information

Manuscript Title: Synchronous Colorectal Adenocarcinomas with Discordant Mismatch Repair Status: A Case of Lynch-like Syndrome and Serrated Pathway Association

Manuscript ID: 1770875

Journal: Frontiers in Oncology

---

1. Title, Keywords and Abstract

- **1a**: The title accurately describes the case type, core clinical features and research focus, and conforms to the norms of case report titles – **Completed**
- **1b**: Relevant retrieval keywords are supplemented to facilitate literature indexing and searching – **Completed**
- **1c**: The abstract covers the research purpose, patient baseline information, key methods, core results and conclusions – **Completed**

2. Introduction

- **2a**: The clinical background and necessity of reporting this case are clearly explained – **Completed**
- **2b**: The unique clinical value and innovative points of this case are clarified – **Completed**

3. Patient Information and Clinical Findings

- **3a**: De-identified patient demographic information (age, gender, occupation) is provided without any privacy information – **Completed (67-year-old female farmer)**
- **3b**: The onset time, development process and main symptoms of the patient are detailed – **Completed**
- **3c**: Past medical history, family history (no hereditary gastrointestinal tumor history), social history are completely recorded – **Completed**
- **3d**: Vital signs, abdominal physical examination, digital rectal examination and

other key physical signs are detailed – **Completed**

- **3e**: Key laboratory tests, imaging and endoscopic results are clearly presented – **Completed**

4. Clinical Assessment and Diagnosis

- **4a**: The complete diagnostic process (imaging, colonoscopy, pathology, molecular detection) is systematically described – **Completed**
- **4b**: The final diagnosis is fully supported by pathological and molecular evidence – **Completed**
- **4c**: Relevant differential diagnoses are considered and excluded – **Completed**

5. Therapeutic Intervention

- **5a**: All treatments (anti-infective therapy, surgery, adjuvant chemoradiotherapy) are detailed – **Completed**
- **5b**: The rationale for surgical and adjuvant treatment decisions is fully explained – **Completed**
- **5c**: Treatments comply with clinical guidelines, and informed consent was obtained from the patient – **Completed**

6. Follow-up and Outcomes

- **6a**: Long-term follow-up time, monitoring items and disease status are recorded (24-month follow-up) – **Completed**
- **6b**: Post-treatment functional recovery, quality of life and clinical prognosis are detailed – **Completed**
- **6c**: Treatment-related adverse reactions and symptomatic management are noted – **Completed**

7. Discussion and Patient Perspective

- **7a**: The case is combined with existing literature to clarify clinical implications and key insights – **Completed**
- **7b**: The patient's treatment experience and personal perspective are included – **Completed**
- **7c**: The limitations of this case report are objectively stated – **Completed**

8. Ethics and Publication Consent

- **8a**: Written informed consent for publication was obtained from the patient – **Completed**

- **8b**: Institutional ethics committee approval was obtained and documented – **Completed**

Author Confirmation: All authors confirm that this manuscript fully complies with the 2020 CARE Guidelines for Case Reports. All checklist items are completed accurately, and all clinical, pathological and molecular information is transparently reported to ensure the standardization and reproducibility of the case report.
